# Towards optimal management of lower eyelid malpositions: A systematic review of treatment effectiveness and safety

**DOI:** 10.1016/j.clinsp.2024.100547

**Published:** 2024-12-03

**Authors:** Lana Sbitan, Haneen Tanous, Mira Nawfal Jardak, Cristina Pires Camargo

**Affiliations:** a Faculty of Medicine, The Hashemite University, Zarqa 13133, Jordan; b Princess Basma Teaching Hospital, Ministry of Health, Irbid, Jordan; c Faculty of Medicine, University of Balamand, Balamand, Lebanon; d Laboratory of Microsurgery and Plastic Surgery (LIM-04), Faculdade de Medicina, Universidade de São Paulo, São Paulo, SP, Brazil

**Keywords:** Ectropion, Entropion, Eyelid diseases, Oculoplastic procedures, Ophthalmic plastic surgery

## Abstract

Lower eyelid malposition, a commonly encountered eyelid disorder, is often linked to discomfort, visual disturbances, and cosmetic concerns. Various treatment modalities have been employed to address these conditions, but their effectiveness and safety remain subjects of debate. This systematic review, aiming to compare and evaluate these treatments, produced a comprehensive analysis of diverse approaches to managing lower eyelid malposition. The search encompassed electronic databases, clinical trial registries, and relevant journals from inception to September 2023. Fifteen studies meeting the inclusion criteria, involving 709 patients (855 eyelids), were reviewed, comprising 12 randomized controlled trials and 3 ongoing trials. We assessed the quality of each included study using the Cochrane Risk-of-Bias tool for randomized trials (RoB v2). We analyzed success rates, recurrence rates, adverse events, and patient satisfaction. Results revealed that modifications to the conventional Lateral Tarsal Strip exhibited high success rates and low recurrence rates. Other treatments, including Everting Sutures, Retraction Pelication, and Orbicularis Muscle Shortening, showed promise in specific cases. Complications, generally mild and manageable, were reported across all modalities, with patients expressing overall satisfaction. The systematic review highlights the effectiveness and safety of various surgical interventions for lower eyelid malposition, offering durable outcomes and minimal recurrence. Tailoring treatments based on patient characteristics and anatomical variations is crucial. Further research is warranted to refine indications for each treatment option in this evolving field.

## Highlights

• A comprehensive systematic review was conducted, to provide healthcare workers with a vital resource in the treatment of eyelid malposition, our search spanned electronic databases, clinical trials registries, and relevant journals.
• The study includes 15 selected trials evaluating 709 patients (855 eyelids), with an assessment of trial quality using the Cochrane Risk-of-Bias tool (RoB V2).

## Introduction

Lower eyelid ectropion and entropion are prevalent eyelid malposition, presenting distinctive challenges in clinical management.^1^ Entropion manifests as the inward rotation of the lid margin, eyelashes, and/or skin against the ocular globe,^1^^,^^2^ while ectropion is characterized by the eversion of the lower lid, exposing the palpebral and bulbar conjunctiva.^1^^,^^3^ These conditions can lead to persistent pain, discomfort, epiphora, conjunctivitis, corneal abrasions, keratitis, and, ultimately, a decrease in visual acuity, resulting in visual impairment.^3^

The management of these malposition's requires identifying their underlying aetiology and understanding the structural anatomy and pathophysiology.^1^ While several surgical interventions have been proposed in the literature as effective treatments, there exists a significant gap in the evidence base regarding their success rates, recurrence rates, and associated adverse events.

This systematic review aims to address this knowledge gap by comprehensively examining the current published evidence on interventions for the correction of lower eyelid malposition's. By analyzing available data, we provide a thorough assessment of the efficacy and safety of various treatment modalities for these debilitating eyelid conditions. This review aims to serve as a vital resource for healthcare professionals seeking evidence-based guidance in the treatment of eyelid malposition, ultimately improving patient outcomes and quality of life.

## Materials and methods

A systematic review was conducted in accordance with the Preferred Reporting Items for Systematic Reviews and Meta-Analysis (PRISMA) guidelines.^4^

### Literature source

The following databases and clinical registries, PubMed, Scopus, Web of Science, Cochrane Central Register of Controlled Trials, Latin American & Caribbean Health Sciences Literature (LILACS), WHO's Clinical Trials Registry Platform (ICTRP), ClinicalTrials.gov, Networked Digital Library of Theses and Dissertations (NDLTD), The ProQuest Dissertations & Theses Global (PQDT), and conference proceedings, were searched using the search strategy: [((entropion* OR ectropion* OR lower eyelids malposition) AND (surgery OR blepharoplast* OR fat graft))].

### Selection criteria

We included clinical trials that reported (I) Individuals who presented with lower eyelid entropion, ectropion, or any type of lower eyelid malposition, (II) Participants of all genders and age categories. We excluded (I) Animal studies, laboratory experiments, in vitro investigations, conference presentations, reviews, and book chapters, as well as (II) Study designs, including case reports, case series, cohort studies, and cross-sectional studies.

The systematic review included search results available at the end of September 2023. The search results were compiled into Endnote software and duplicates were identified and removed.

During the initial screening, authors (L.S and M.N.J) independently reviewed articles based on titles and abstracts. Disagreements were resolved through discussion with a third reviewer, (C.P.C.) In the subsequent screening, authors (C.P.C, L.S, M.N.J, and H.T) independently reviewed full articles in pairs. Disagreements were resolved through discussion of the studies in question. The bibliographies of the studies included in the review were subsequently reviewed to identify any further relevant studies for inclusion.

### Data extraction

We developed a data extraction form and two independent authors (L.S and H.T) collected information from each eligible study, including reference details (first author and publication year), study design, number of patients, age, gender, intervention, control, intervention success, recurrence (6-months to 2-years post-intervention), patient satisfaction, and major post-intervention adverse events (e.g., external rotation, epiphora, eye irritation, corneal ulceration, scarring, wound dehiscence, eyelid asymmetry, and infections).

Due to the heterogeneity of the included studies, a meta-analysis was not applicable. Consequently, the findings of the included studies were compared descriptively.

### Quality assessment

Two independent reviewers (L.S and H.T) employed the Cochrane Risk-of-Bias tool for randomized trials (RoB v2) to conduct quality assessments of the included studies.^5^ This tool evaluated the randomization process, deviations from intended interventions, missing outcome data, outcome measurement, and selection of the reported results.

## Results

### Literature source

**Fig. 1:**
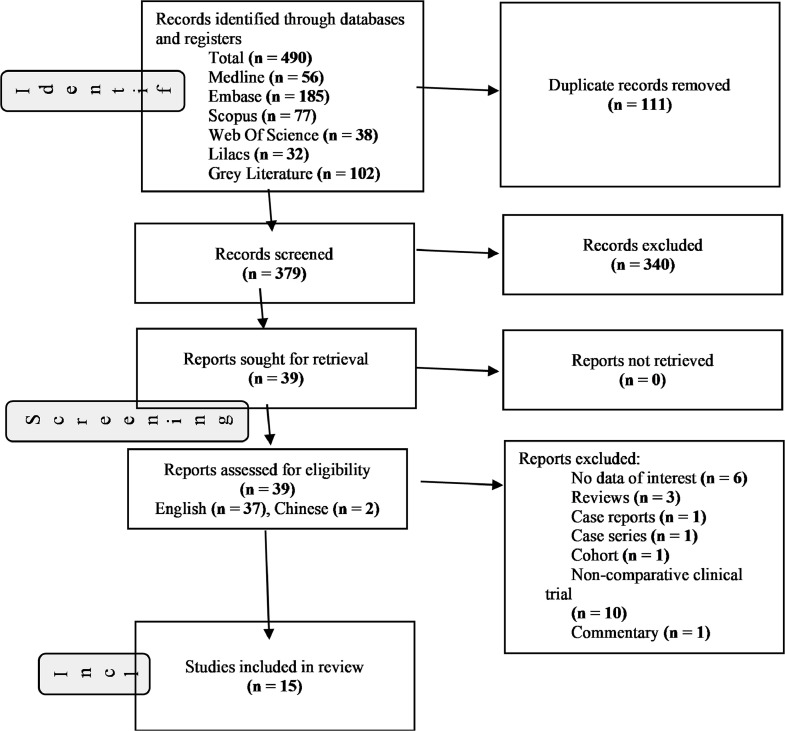
Flow diagram summarizing study selection process. This flow diagram illustrates the systematic selection process of studies included in the review.

The electronic database search yielded 490 records. After eliminating duplicate entries, we preliminarily screened based on titles and abstracts, resulting in 379 studies. Out of these, 340 records did not meet our inclusion criteria. We obtained full-text copies of 39 studies for further assessment. Following a joint review by all authors, 24 studies were excluded, leaving 12 studies^3^^,^^6^, ^7^, ^8^, ^9^, ^10^, ^11^, ^12^, ^13^, ^14^, ^15^, ^16^ and 3 ongoing clinical trials that met the criteria^17^, ^18^, ^19^ (refer to Fig. 1).

### Quality assessment of the included studies

The overall quality assessment of the 12 randomized clinical trials included in this study,^3^^,^^6^, ^7^, ^8^, ^9^, ^10^, ^11^, ^12^, ^13^, ^14^, ^15^, ^16^ conducted using the Cochrane Risk-of-Bias tool (ROB v2), revealed the following:

- Four of the included studies were assessed as having a high risk of bias.^3^^,^^9^^,^^11^^,^^12^
- Three trials were categorized as having an unclear risk of bias.^6^^,^^7^^,^^16^
- Five reports were deemed to have a low risk of bias.^8^^,^^10^^,^^13^, ^14^, ^15^

In the randomization process domain, two clinical trials were categorized as having an unclear risk,^10^^,^^11^ although most studies showed a low risk.^3^^,^^6^^,^^7^^,^^9^^,^^12^, ^13^, ^14^, ^15^, ^16^ Concerning deviation from intended interventions, two studies were found to have a high risk of bias,^9^^,^^11^ while three trials had an unclear risk.^6^^,^^7^ With regard to missing data, only one study was considered as having a high risk of bias.^12^ In the domain of outcome measurement, the risk of bias was unclear in most clinical trials (seven out of the twelve).^3^^,^^6^^,^^7^^,^^12^, ^13^, ^14^^,^^16^ As for selection bias, three of the included randomized clinical trials were determined to have a high risk^3^^,^^11^^,^^12^ (refer to Supplementary Table 1).

### Ongoing clinical trials

Three active clinical trials were identified through searches in clinical trial registries, as depicted in Online Resource 1 (Supplementary Table 2).^17^, ^18^, ^19^

### Demographics and publication trends

Our systematic review included 709 patients, with 855 operated eyelids, aged 50 to 80 years. Most studies excluded bilateral lower eyelid malposition,^6^, ^7^, ^8^, ^9^, ^10^, ^11^, ^12^, ^13^, ^14^^,^^16^ except for two studies.^3^^,^^15^ Gender distribution among patients in the included studies was roughly an equal ratio of females to males.

Only one clinical trial included in this review was published in 1998,^13^ while 11 studies were published after the year 2003.^6^, ^7^, ^8^, ^9^, ^10^, ^11^, ^12^, ^13^, ^14^^,^^16^ Among them, three clinical trials were conducted in Italy,^6^^,^^7^^,^^11^ two in the UK,^13^^,^^14^ two in China,^15^^,^^16^ and the others in Spain,^3^ India,^9^ Greece,^12^ Germany,^8^ and Pakistan.^10^ Table 1 outlines the demographic and baseline characteristics of the included studies (refer to Table 2).

**Table 1:** Summary of studies comparing approaches for treating various forms of lower eyelid malposition.

Study ID	Intervention	Control	Outcome Measures	Outcomes								Follow-up duration
				Intervention Success		Recurrence		Adverse Events		Patient Satisfaction		
				Intervention	Control	Intervention	Control	Intervention	Control	Intervention	Control	
Olver et al. 1998^1^	Donor scleral graft interposition without adjuvant antimetabolites	Partial tenotomy of the Anterior Lower Eyelid Retractors (ALER), capsulopalpebral fascia, and inferior tarsal muscle, with a single preoperative application of adjuvant 5-fluorouracil or mitomycin C	Lower margin reflex distance (MRD): distance (in mm) from the central corneal light reflex to the lower eyelid margin.	Significant lower MRD reduction (mean 1.8 mm; *p* < 0.001) and improved eyelid contour at 1, 3, 6 months post-surgery. Lower SS significantly reduced at 1, 3, 6 months after surgery (*p* < 0.001).	Significant lower MRD reduction at 1-month post-surgery (*p* = 0.006), not sustained at 3 and 6 months (*p* = 0.16 and 0.10). Significant lower SS reduction at 1-month post-surgery (*p* < 0.001), not sustained at 3 and 6 months (*p* = 0.12 and 0.22). No significant differences between patients treated with adjuvant 5-fluorouracil or mitomycin C.	None of the patients required surgery for recurrence.	3 patients (4 eyelids) required surgery for recurrent retraction; all received mitomycin C. 4 patients (5 eyelids) required surgery for recurrent central sag in the eyelid contour.	Results were satisfactory in all patients.	Results were satisfactory only in 5 patients (6 eyelids).	None reported.	Unilateral corneal epithelial erosion (1 patient). Temporary adhesion of lower eyelid to bulbar conjunctiva (1 patient).	7.8 months Fixed intervals (1-week and at 1, 3, 6, 9, 12, and 18 months) after surgery.
			Lower scleral show (SS): distance from the inferior limbus to the lower eyelid margin.									
López-García et al. 2017^2^	Modified tarsal strip technique (Suture placement modified for entropion and ectropion)	Conventional lateral tarsal strip	Horizontal lid laxity (HLL) measured by pinch test, orbicularis muscle function and hypertrophy, lower eyelid retractors function.	All ectropion patients achieved successful surgical outcomes. 48 of 50 entropion eyelids had successful surgical outcomes (96%). Orbicularis hypertrophy (8 eyelids). Statistically significant HLL and orbicularis muscle function compared to the control group (*p* < 0.05).	Mean HLL in entropion eyelids = 5.8; in ectropion patients = 5.8. Orbicularis hypertrophy (12 eyelids) Lower eyelid retractors' dysfunction noted in 22 eyelids.	2 out of 94 eyelids (4%) showed recurrence. (entropion)	9 out of 90 eyelids showed recurrence (17.4% entropion and 2.3% ectropion)	No difference in patient perception was found between groups. Subjective perception significantly improved after treatment (*p* = 0.001).	No difference in postoperative complications between groups. Moderate to severe lower lid hematomas were observed in 10 eyelids. Ectropion overcorrection (9 eyelids). Entropion overcorrection (8 eyelids).	64.8 months. Fixed intervals (1, 10, 30 days, 6, 12 months, 2, 3, 4, 5, 6 years) post-surgery.		

1. Olver JM, Rose GE, Khaw PT, Collin JR. Correction of lower eyelid retraction in thyroid eye disease: a randomized controlled trial of retractor tenotomy with adjuvant antimetabolite versus scleral graft. Br J Ophthalmol. 1998;82(2):174–80.

2. López-García JS, García-Lozano I, Giménez-Vallejo C, Jiménez B, Sánchez Á, de Juan IE. Modified lateral tarsal strip for involutional entropion and ectropion surgery. Graefes Arch Clin Exp Ophthalmol Albrecht Von Graefes Arch Klin Exp Ophthalmol. 2017;255(3):619–25.

**Table 2:** Demographics and baseline characteristics of the included studies.

Study ID	Country	Study design	Sample Size (number of eyelids)			Age (mean 'years')			Gender (Females 'F'|Males 'M')		
			Total	Intervention	Control	Total	Intervention	Control	Total	Intervention	Control
Olver et al. 1998^1^	UK	RCT	25 (35)	13 (20)	12 (15)	57	54	59	20F|5M	9F|4M	11F|1M
Altieri et al. 2003^2^	Italy	RCT	43 (43)	13 (13)	Group 2: 16 (16)	75.7	70.9	Group 2: 78.7	31F|12M	8F|5M	Group 2: 14F|2M
					Group 3: 14 (14)			Group 3: 71.8			Group 3: 11F|3M
Altieri et al. 2004^3^	Italy	RCT	69 (69)	33 (33)	36 (36)	74.8	77.5	70.3	62^a^ (39F|23M)	15F|13M	24F|10M
Scheepers et al. 2010^4^	UK	RCT	63 (63)	26 (26)	29 (29)	77	78	77	28F|27M	13F|13M	15F|14M
			55 (55)^a^								
Xu, 2015^5^	China	RCT	82 (126)	41 (63)	41 (63)	67.3	68.8	67.7	26F|56M	11F|30M	15F|26M
López-García et al. 2017^6^	Spain	RCT	135 (184)	(94 eyelids)	(90 eyelids)	NA	71.2	67.4	93F|91M (eyelids)	NA	NA
			110^a^								
Goel et al. 2017^7^	India	RCT	30 (30)	15 (15)	15 (15)	Range (55‒80 years)	69.33	70.53	11F|19M	NA	NA
Meduri et al. 2018^8^	Italy	RCT	8 (16)	4 (8)	4 (8)	69.5	71	NA	NA	NA	NA
Nakos et al. 2019^9^	Greece	RCT	45 (54)	(28 eyelids)	(26 eyelids)	72.69	72	72.8	28F|26M (eyelids)	15F|13M (eyelids)	13F|13M (eyelids)
Dulz et al. 2019^10^	Germany	RCT	52 (66)	(30 eyelids)	(36 eyelids)	73	65.4	67.2	NA	NA	NA
			49 (63)^a^								
Yuan, 2020^11^	China	RCT	79 (91)	41 (46)	38 (45)	73.1	73.2	72.9	39F|40M	20F|21M	19F|19M
Khan et al. 2021^12^	Pakistan	RCT	78 (78)	39 (39)	39 (39)	Range (55‒80 years)	64.62	64.56	23F|55M	NA	NA

a Excluding patients lost to follow-up.

1. Olver JM, Rose GE, Khaw PT, Collin JR. Correction of lower eyelid retraction in thyroid eye disease: a randomised controlled trial of retractor tenotomy with adjuvant antimetabolite versus scleral graft. Br J Ophthalmol. 1998;82(2):174–80.

2. Altieri M, Iester M, Harman F, Bertagno R, Capris P, Venzano D, et al. Comparison of three techniques for repair of involutional lower lid entropion: a three-year follow-up study. Ophthalmol J Int Ophtalmol Int J Ophthalmol Z Augenheilkd. 2003;217(4):265–72.

3. Altieri M, Kingston AEH, Bertagno R, Altieri G. Modified retractor plication technique in lower lid entropion repair: a 4-year follow-up study. Can J Ophthalmol J Can Ophtalmol. 2004;39(6):650–5.

4. Scheepers MA, Singh R, Ng J, Zuercher D, Gibson A, Bunce C, et al. A randomized controlled trial comparing everting sutures with everting sutures and a lateral tarsal strip for involutional entropion. Ophthalmology. 2010;117(2):352–5.

5. Xu QL. Clinical efficacy comparison of flabby skin excision combined orbicularis oculi muscle shortening surgery in patients with senile entropion. Int Eye Sci. 2015;15:1277–9.

6. López-García JS, García-Lozano I, Giménez-Vallejo C, Jiménez B, Sánchez Á, de Juan IE. Modified lateral tarsal strip for involutional entropion and ectropion surgery. Graefes Arch Clin Exp Ophthalmol Albrecht Von Graefes Arch Klin Exp Ophthalmol. 2017;255(3):619–25.

7. Goel R, Sanoria A, Kumar S, Arya D, Nagpal S, Rathie N. Comparison of Polypropylene Sling with Combined Transconjunctival Retractor Plication and Lateral Tarsal Strip for Correction of Involutional Lower Eye Lid Ectropion. Open Ophthalmol J. 2017;11:285–97.

8. Meduri A, Inferrera L, Oliverio GW, Tumminello G, Rechichi M, Mazzotta C, et al. The Use of a Double Suture and Conjunctival Cuts in the Lateral Tarsal Strip: A New Approach to Involutional Ectropion. J Craniofac Surg. 2018;29(8):2312–5.

9. Nakos EA, Boboridis KG, Kakavouti-Doudou AA, Almaliotis DD, Sioulis CE, Karampatakis VE. Randomized Controlled Trial Comparing Everting Sutures with a Lateral Tarsal Strip for Involutional Lower Eyelid Entropion. Ophthalmol Ther. 2019;8(3):397–406.

10. Dulz S, Green S, Mehlan J, Schüttauf F, Keserü M. A comparison of the lateral tarsal strip with everting sutures and the Quickert procedure for involutional entropion. Acta Ophthalmol (Copenh). 2019;97(6):e933–6.

11. Yuan W. Treatment of degenerative entropion with lower eyelid muscle reduction and lateral tarsal plate fixation. Int Eye Sci. 2020;2181–4.

12. Khan FA, Hyder MF, Khan Niazi SP, Mirza UT. Comparison of the Recurrence Rate of Entropion via Subciliary Route and Transconjunctival Route in Combined Lateral Tarsal Strip with Retractor Advancement Procedure for Involutional Entropion Correction. J Coll Physicians Surg ‒ Pak JCPSP. 2021;30(4):429–33.

### Lower eyelid malposition treatment

This study included two clinical trials investigating treatment options for various types of Lower Eyelid Malposition (LEM), including both Entropion and Ectropion.^3^^,^^13^ Olver et al. compared the effectiveness of scleral graft interposition with partial tenotomy of the Anterior part of the Lower Eyelid Retractors (ALER).^13^ Lopez-Garcia et al. investigated a modified technique for the lateral tarsal strip procedure in treating both ectropion and entropion.^3^ Olver et al. also explored the impact of preoperative application of adjuvant antimetabolites, such as 5-fluorouracil or mitomycin C, in treating lower eyelid malposition.^13^

Analysis of success rate

In Olver et al., both the scleral graft interposition and partial tenotomy groups experienced a statistically significant reduction in lower Margin Reflex Distance (MRD) and lower Scleral Show (SS) (p-value < 0.05).^13^ However, reduction post-partial tenotomy was not sustained at 3 or 6 months, unlike scleral graft interposition.^13^ No significant difference in the change of lower MRD and SS between patients treated with adjuvant antimetabolites 5-fluorouracil or mitomycin C.^13^

The modified tarsal strip technique demonstrated successful outcomes, as indicated by statistically significant improvements in Horizontal Lid Laxity (HLL) and orbicularis muscle function (p-value < 0.05) compared to the control group (conventional lateral tarsal strip).^3^

Analysis of recurrence rate

Patients with donor scleral graft interposition, without adjuvant antimetabolites, didn't need further surgery. In contrast, 7 patients (9 lower eyelids) with partial tenotomy of ALER experienced post-surgery recurrence.^13^ The modified tarsal strip technique resulted in a 4% entropion recurrence rate, while the conventional lateral tarsal strip group had entropion and ectropion recurrence rates of 17.4% and 2.3%, respectively.^3^

Analysis of adverse events

No significant complications differed between the modified tarsal strip and conventional groups.^3^ However, among patients with partial tenotomy of ALER, two experienced unilateral corneal epithelial erosion (1 patient) and temporary adhesion between the lower eyelid and the bulbar conjunctiva (1 patient).^13^

Analysis of patients' satisfaction

Only five patients who had undergone partial tenotomy of ALER reported unsatisfactory results^13^ (Table 1).

### Ectropion treatment

In our review, two clinical trials focused on treating ectropion using four distinct approaches.^9^^,^^11^ Meduri et al. compared a modified technique for the Lateral Tarsal Strip to the conventional Lateral Tarsal Strip.^11^ Goel et al. assessed the effectiveness of Transconjunctival Retractor Plication combined with the Lateral Tarsal Strip, comparing it to the polypropylene sling.^9^

Analysis of success rate

No statistical significance in the success rate was observed between Transconjunctival Retractor Plication combined with the Lateral Tarsal Strip and polypropylene sling groups (p-value = 0.271).^9^ In contrast, a statistically significant difference was noted by Meduri et al. between the intervention and control groups (p-value < 0.05).^11^

Both studies conducted post-surgical patient follow-up, one following up for 12 months,^9^ and the other for 18 months.^11^

Analysis of recurrence rate

While Meduri et al. reported no observed recurrence following interventions,^11^ Goel et al. did not analyze the post-surgery recurrence rate.^9^

Analysis of adverse events

Following polypropylene sling use, lateral ectropion on the first day postoperatively (one eyelid), and Grade 1 lateral entropion at the 5th-month follow-up (one eyelid) were observed.^9^ In contrast, no complications were reported for the Transconjunctival retractor plication with lateral tarsal strip intervention.^9^ Despite a lack of statistical significance (*p* = 0.072), no adverse events occurred with the Modified and Conventional lateral tarsal strip procedures.^11^

Analysis of patients' satisfaction

The domain of patient satisfaction was not analyzed in either study (Table 3).^9^^,^^11^

**Table 3:** Overview of clinical trials comparing approaches for ectropion treatment.

Study ID	Intervention	Control	Outcome Measures	Outcomes								Follow-up duration
				Intervention Success		Recurrence		Adverse Events		Patient Satisfaction		
				Intervention	Control	Intervention	Control	Intervention	Control	Intervention	Control	
Goel et al. 2017^1^	Transconjunctival retractor plication with lateral tarsal strip	Polypropylene sling	Success is defined as no epiphora and lid laxity of 4 mm or less at 12-month follow-up. Eyelid laxity was assessed through pinch test, lateral distraction test for medial canthal tendon laxity, lateral canthal tendon laxity, inferior lid retractor laxity, and position of puncta on upgaze and primary gaze. Ectropion graded using Moe and Linder scale.	Success rate: 14/15 eyelids (93.33%). No statistically significant difference in success rates between groups (*p* = 0.271).	Success rate: 13/15 eyelids (87%)	NA	NA	NA	NA	None reported. Complication occurrences between the two groups showed no statistically significant difference (*p* = 0.072).	Lateral ectropion observed on the 1st postoperative day, lasting ten days. Subsequently, a return to initial preoperative laxity confirmed at the 4th-week follow-up (1 eyelid). Grade 1 lateral entropion (inner margin inturned) developed at the 5th-month follow-up (1 eyelid).	12 months
Meduri et al. 2018^2^	Modified lateral tarsal strip	Conventional lateral tarsal strip	Success is defined as relief of lid laxity. HLL by pinch test.	Mean HLL: 3.5 mm. Statistically significant difference was observed between intervention and control groups (*p* < 0.05).	Mean HLL = 5.7 mm	None observed	None observed	NA	NA	None reported	None reported	18 months Fixed intervals (1, 6, 12, 18 months) post-intervention.

1. Goel R, Sanoria A, Kumar S, Arya D, Nagpal S, Rathie N. Comparison of Polypropylene Sling with Combined Transconjunctival Retractor Plication and Lateral Tarsal Strip for Correction of Involutional Lower Eye Lid Ectropion. Open Ophthalmol J. 2017;11:285–97.

2. Meduri A, Inferrera L, Oliverio GW, Tumminello G, Rechichi M, Mazzotta C, et al. The Use of a Double Suture and Conjunctival Cuts in the Lateral Tarsal Strip: A New Approach to Involutional Ectropion. J Craniofac Surg. 2018;29(8):2312–5.

### Entropion treatment

The remaining studies included in this review addressed Entropion treatment through broader surgical techniques.^6^, ^7^, ^8^^,^^10^^,^^12^^,^^14^, ^15^, ^16^

Everting Sutures (ES)

Concerning ES as a treatment for entropion, Altieri et al. compared ES to two alternative approaches.^6^ One group used a modified technique, involving Retractor Plication and partial resection of the pretarsal orbicularis, while the other underwent horizontal lid tightening at the lower tarsal border.

Conversely, Scheepers et al. investigated horizontal eyelid shortening through the lateral tarsal strip procedure in conjunction with ES.^14^ Nakos et al. and Dulz et al. conducted comparative studies involving ES and the Lateral Tarsal Strip Procedure (LTS) (Table 4).^8^^,^^12^

**Table 4:** Summary of clinical trials focused on entropion treatment.

Study ID	Intervention	Control	Outcome Measures	Outcomes								Follow-up duration
				Intervention Success		Recurrence		Adverse Events		Patient Satisfaction		
				Intervention	Control	Intervention	Control	Intervention	Control	Intervention	Control	
Clinical trials focused on Entropion Treatment using Everting Sutures (ES)												
Altieri et al. 2003^1^	Retractor plication and partial resection of pretarsal orbicularis (a modified technique)	Group 2: Everting sutures.	HLL: pinch test.	Statistically significant difference in preoperative and postoperative LLE and HLL measurements (*p* < 0.05)	Group 2: No significant difference between preoperative and postoperative HLL (*p* > 0.05) Significant difference in preoperative and postoperative LLE measurements (*p* < 0.05)	2/9 patients had recurrence (22.2%)	Group 2: 4/13 patients had recurrence.	NA	NA	NA	NA	36 months Fixed intervals (1, 6 months, 1, 2 and 3 years) post-operatively. (32 patients)
		Group 3: Fox Procedure (horizontal tightening of lid at lower tarsal border)	Lateral tarsal strip (LLE): measure vertical excursion in millimeters of the lower lid margin center between extreme upgaze and downgaze.		Group 3: No significant difference in preoperative and postoperative measurements of LLE and HLL (*p* > 0.05)		Group 3: 3/10 patients had recurrence.					
							Statistically significant difference in recurrence rates between Group 2 and Group 3 (*p* < 0.05).					
Scheepers et al. 2010^2^	Everting suture and horizontal eyelid shortening using lateral tarsal strip procedure.	Everting suture	HLL: pinch test.	Mean HLL: 9.5 mm. No statistically significant difference between intervention and control groups (*p* < 0.005).	Mean HLL: 9.6 mm.	0/26 patients had recurrence (0%). Statistically significant difference between intervention and control groups (*p* = 0.024).	6/29 patients had recurrence (21%).	None reported.	Suture-related granuloma (2 patients).	NA	NA	18 months Fixed intervals (3 weeks, 6, 12 and 18 months) post-operatively (55 patients)
Nakos et al. 2019^3^	Quickert everting sutures	Lateral tarsal strip	Success: Full anatomical restoration of eyelid position and symptom resolution. National Eye Institute Visual Functioning Questionnaire-25 (NEI VFQ-25) was used to assess function and symptoms.	Statistically significant difference: LTS procedure alone more effective than ES technique at 12-month follow-up (*p* = 0.015).	40 eyelids had recurrence across all follow-up visits.	8 eyelids had recurrence across all follow-up visits. The LTS group showed significantly lower recurrence rates compared to the ES group at both follow-ups, with statistical significance (*p* = 0.025 and 0.015).	None reported.	Abscess in lateral canthal area at 12 months, possibly from polyethylene fixation suture granuloma (1 patient).	NA	NA	36 months Fixed intervals (1 week, 6 and 12 months) post-operatively.	
Dulz et al. 2019^4^	Lateral tarsal strip with Everting sutures	Quickert everting sutures	Success: Lower eyelid consistently contacts ocular surface in both relaxed and intentionally closed states, with no entropion or ectropion symptoms.	Success rate: 27/30 eyelids (93.10%). No statistically significant difference in surgical failure between intervention and control groups (*p* = 0.46).	Success rate: 33/36 (97.06%).	2/30 eyelids	1/36 eyelids	Postoperative secondary ectropion (1 patient)	Postoperative secondary ectropion (2 patients)	NA	NA	14 months Fixed Intervals (2 weeks, 8 and 14 months) post-operatively.
Clinical trials Focused on Entropion Treatment using Lateral Tarsal Strip (LTS)												
Yuan 2020^5^	Lower eyelid constrictor muscle reduction with lateral tarsal strip	Lower eyelid constrictor muscle reduction	Short-term cure: 3 months post-surgery. Long-term cure: 24 months post-surgery. Measure positive rates of square pulling test and lower eyelid repositioning test.	Short-term cure rate: 100%. Long-term cure rate: 98%. No statistically significant difference in short-term cure rate (*p* = 0.495). Long-term cure rate in the intervention group was higher than in the control group (*p* = 0.030).	Short-term cure rate: 98%. Long-term cure rate: 84%.	NA	NA	None reported. The intervention group had fewer postoperative complications than the control group, with statistical significance (*p* = 0.026).	Postoperative complications rate = 11%. Eyelid ectropion (2 eyelids). Scleroderma (3 eyelids).	NA	NA	24 months Fixed Intervals (1, 7, 30 days and 3, 12, 24 months) post-operatively.
Khan et al. 2021^6^	Lateral tarsal strip procedure, combined with retractor advancement, through the subciliary approach	Lateral tarsal strip procedure, combined with retractor advancement, through the transconjunctival approach	Success: restoration of normal anatomical position of lid margin without inward turning of lid margin on eyelid closure.	NA	NA	Recurrence rate: 2.6%	Recurrence rate: 5.1% No statistically significant difference in entropion recurrence between transconjunctival and subciliary route repairs (*p* > 0.999).	Ectropion (2 patients), stitch abscess (1 patient), lateral canthal dystopia (2 patients), visible scar in the infraciliary region (3 patients).	Mild conjunctivochalasis (3 patients). Lateral canthal dystopia (1 patient).	NA	NA	12 months Fixed intervals (1, 3, 6 and 12 months) post-operatively.
Clinical trials focused on Entropion Treatment using either Retractor Plication (RP) or Orbicularis Muscle Shortening (OMS)												
Altieri et al. 2004^7^	Modified retractor plication technique	Jones retractor plication technique	HLL: pinch test. Medial canthal tendon laxity	Mean HLL: 6.86 mm. Mean medial canthal tendon laxity at rest = 1.90 mm. Significantly less horizontal lid laxity and medial canthal tendon laxity in the modified technique group compared to the Jones technique group (*p* < 0.05).	Mean HLL: 7.30 mm. Mean medial canthal tendon laxity at rest: 1.25 mm.	2/28 patients had recurrence (7.1%) Statistically significant difference between intervention and control groups (*p* < 0.05).	5/34 patients had recurrence (14.7%).	NA	NA	NA	NA	48 months Fixed intervals (1, 6 months, 1 year, 2, 3 and 4 years) post-operatively. (62 patients)
Xu 2015^8^	Loose skin excision and shortening of the orbicularis muscle	Shortening of the orbicularis oculi muscle without excision of loose skin	Short-term cure: Following one month of treatment, normal eyelid and lacrimal point positions in open and closed-eye conditions, normal eyelashes, no epiphora or irritation symptoms, and avoiding touching the eye. Long-term cure: No irritation symptoms in the cornea and conjunctiva after a 1.5-year follow-up. Efficiency calculated as (cured eyes + normal eyes) / total eyes.	Short-term effective rate: 95.2%. Long-term cure rate: 82.5%. Statistically significant differences in short-term and long-term cure rates between intervention and control groups (p-value = 0.043, < 0.05) respectively.	The short- term effective rate: 77.8%. The long-term cure rate: 39.7%	7(11)/41(63) patients had recurrence (17.5%).	16(25)/ 41(63) patients had recurrence (39.7%).	NA	NA	NA	NA	18 months

1. Altieri M, Iester M, Harman F, Bertagno R, Capris P, Venzano D, et al. Comparison of three techniques for repair of involutional lower lid entropion: a three-year follow-up study. Ophthalmol J Int Ophtalmol Int J Ophthalmol Z Augenheilkd. 2003;217(4):265–72.

2. Scheepers MA, Singh R, Ng J, Zuercher D, Gibson A, Bunce C, et al. A randomized controlled trial comparing everting sutures with everting sutures and a lateral tarsal strip for involutional entropion. Ophthalmology. 2010;117(2):352–5.

3. Nakos EA, Boboridis KG, Kakavouti-Doudou AA, Almaliotis DD, Sioulis CE, Karampatakis VE. Randomized Controlled Trial Comparing Everting Sutures with a Lateral Tarsal Strip for Involutional Lower Eyelid Entropion. Ophthalmol Ther. 2019;8(3):397–406.

4. Dulz S, Green S, Mehlan J, Schüttauf F, Keserü M. A comparison of the lateral tarsal strip with everting sutures and the Quickert procedure for involutional entropion. Acta Ophthalmol (Copenh). 2019;97(6):e933–6.

5. Yuan W. Treatment of degenerative entropion with lower eyelid muscle reduction and lateral tarsal plate fixation. Int Eye Sci. 2020;2181–4.

6. Khan FA, Hyder MF, Khan Niazi SP, Mirza UT. Comparison of the Recurrence Rate of Entropion via Subciliary Route and Transconjunctival Route in Combined Lateral Tarsal Strip with Retractor Advancement Procedure for Involutional Entropion Correction. J Coll Physicians Surg Pak. 2021;30(4):429–33.

7. Altieri M, Kingston AEH, Bertagno R, Altieri G. Modified retractor plication technique in lower lid entropion repair: a 4-year follow-up study. Can J Ophthalmol J Can Ophtalmol. 2004;39(6):650–5.

8. Xu QL. Clinical efficacy comparison of flabby skin excision combined orbicularis oculi muscle shortening surgery in patients with senile entropion. Int Eye Sci. 2015;15:1277–9.

Analysis of success rate

In the first study, HLL showed no statistically significant difference pre- and postoperatively in the ES group (*p* > 0.05), while Lower Lid Excursion (LLE) demonstrated a significant difference (*p* < 0.05).^6^

Scheepers et al. found no statistically significant difference between the intervention and control groups (*p* < 0.005).^14^ Similarly, Dulz et al. reported no statistically significant difference in surgical failure between the LTS with ES group and the Quickert ES group (*p* = 0.46).^8^ In contrast, Nakos et al. identified a statistically significant difference in success rates between the LTS and ES groups (p-value = 0.015).^12^

Analysis of recurrence rate

Of the 13 patients who underwent the ES procedure, Altieri et al. reported four cases of recurrence,^6^ while Scheepers et al. observed a 21% recurrence rate.^14^ Dulz et al. reported two entropion recurrence cases,^8^ and Nakos et al. documented 40 eyelid recurrences.^12^

Analysis of adverse events

In Scheepers et al.'s study, two patients experienced suture-related granuloma.^14^ Additionally, Nakos et al. reported one incidence of abscess development in the lateral canthal area 12-months after surgery in the LTS group.^12^ Similarly, Dulz et al. reported postoperative secondary ectropion.^8^ Notably, Altieri et al. did not analyze this domain.^6^

Analysis of patients' satisfaction

None of these studies assessed patient satisfaction following surgery (Table 4).^6^^,^^8^^,^^12^^,^^14^

Lateral Tarsal Strip (LTS)

Analysis of success rate

Khan et al. compared the subciliary approach and the transconjunctival approaches in the LTS procedure but did not report success rates.^10^

Yuan explored the incorporation of lower eyelid constrictor muscle reduction into the LTS procedure, finding no statistically significant difference in the short-term curative rate (p-value = 0.495).^16^ However, in the long term, the lower eyelid constrictor muscle reduction with the LTS group showed a higher curative rate than the control group (p-value = 0.030) (Table 4).^16^

Analysis of recurrence rate

The recurrence rate of entropion following repair via the transconjunctival route was not found to be statistically significant when compared to the subciliary route (p-value > 0.999).^10^ Yuan didn't analyze the recurrence rate following an intervention.^16^

Analysis of adverse events

Yuan's study demonstrated that the intervention group experienced significantly fewer postoperative complications compared to the control group (p-value = 0.026).^16^ However, eight out of 39 patients who underwent the subciliary approach in Khan et al.'s study reported postoperative complications, including ectropion, stitch abscess, lateral canthal dystopia, and a visible scar in the infraciliary region.^10^

Analysis of patients' satisfaction

Neither of the studies assessed the domain of patient perception post-intervention.^10^^,^^16^

Retractor Plication (RP)

Analysis of success rate

Altieri et al. noted a statistically significant reduction in HLL and medial canthal tendon laxity in the group that underwent a modified RP technique, compared to the Jones RP technique (p-value < 0.05) (Table 4).^7^

Analysis of recurrence rate

The modified RP group reported a 7.1% recurrence rate.^7^

Analysis of adverse events

Postoperative complications were not analyzed in this study.^7^

Analysis of patients' satisfaction

Altieri et al. did not assess patient satisfaction after surgery.^7^

Orbicularis Muscle Shortening (OMS)

Analysis of success rate

Xu observed significant differences in short-term and long-term cure rates between the group with loose skin excision alongside orbicularis oculi muscle shortening and the control group without excision (*p* < 0.05) (Table 4).^15^

Analysis of recurrence rate

Patients with loose skin excision alongside OMS had a lower recurrence rate (17.5%) compared to the control group with a recurrence rate of 39.7%.^15^

Analysis of adverse events

Xu did not assess postoperative complications following interventions.^15^

Analysis of patients' satisfaction

Patient's satisfaction domain was not analyzed in this study.^15^

## Discussion

In this systematic review, we included 12 studies,^3^^,^^6^, ^7^, ^8^, ^9^, ^10^, ^11^, ^12^, ^13^, ^14^, ^15^, ^16^ describing 709 patients (855 eyelids) that underwent various treatment modalities for the correction of lower eyelid malposition, with a focus on the safety and efficacy of these interventions. We initiate the discussion with a review of the treatment options explored thus far.

Ectropion and entropion are either congenital or acquired.^1^ Acquired ectropion may result from mechanical, paralytic, cicatricial, or involutional causes, while acquired entropion can be acute spastic, cicatricial, or involutional.^1^ Management options can range from conservative to surgical, for the sake of relevance to the systematic review, we will focus on the surgical interventions employed.

Cicatricial ectropion, marked by scar tissue causing vertical shortening of the anterior or middle lamella,^1^^,^^20^, ^21^, ^22^, ^23^ may be heightened by aggressive transcutaneous lower lid blepharoplasty, elevating the risk.^20^^,^^22^ Surgical options involve lower eyelid tightening and full-thickness spacer grafts for anterior lamella shortening.^9^^,^^20^^,^^22^ Paralytic ectropion management depends on its duration; surgical procedures include horizontal lid tightening, canthoplasty, spacer grafts, and retractor release with support using silicone sling or fascia lata, and tarsorrhaphies.^1^^,^^22^ Mechanical ectropion treatment targets the underlying disease with surgical interventions addressing lower lid laxity after mass excision and eyelid reconstruction.^1^^,^^22^

Treatment for Cicatricial entropion involves repositioning lashes, restoring posterior lamellar height through scar lysis, mucosal spacer grafts, suturing techniques, blepharotomy, and tarsal fractures.^20^^,^^22^ Managing Spastic entropion includes eyelid sutures or botulinum toxin injections to ease muscle hyperactivity.^1^ Involutional entropion and ectropion, common in clinical practice, notably amongst patients over 60 years old,^23^ have distinct pathophysiological factors, including horizontal lid laxity, retractor disinsertion, and muscle changes.^1^^,^^20^^,^^22^ Surgical corrections vary by etiology, with procedures such as lateral tarsal strip, canthopexy, wedge resection, and retinacular canthoplasty addressing specific age-related changes.^20^^,^^22^

As described above, various surgical methods have been considered in the literature with variable success rates to address lower eyelid malposition, a successful outcome is often reached only after a combination of treatment modalities. Complete surgical success was found in patients who underwent combined, multiple-modality surgical interventions, such as the modified lateral tarsal strip. In contrast, anatomical and functional recurrence was only evident in patients who underwent a more conventional, single-modality treatment, regardless of what the modality was.

A comparable review published in 2011 by Boboridis & Bunce, included a single Randomized Clinical Trial (RCT),^14^ which concluded that the combined use of everting sutures and lateral tarsal strips for both horizontal and vertical eyelid tightening is more effective in treating involutional entropion than vertical tightening with everting sutures alone.^24^ Our review expanded on this, comparing it to three other trials all involving everting sutures. In terms of success rate, Scheepers et al. and Dulz et al. reported no significant difference in their comparative groups.^8^^,^^14^ Conversely, Nakos et al. noted a significant difference in success rates between the LTS and ES groups (p-value = 0.015), favoring the LTS group.^12^

Our systematic review is intended as a crucial reference, offering evidence-based insights to guide healthcare professionals in treating eyelid malposition, with the ultimate goal of enhancing patient outcomes and overall quality of life. The data from the clinical trials included in this review was presented and compared to facilitate meaningful inferences. Further evidence in the form of RCTs will aid us in making a firm recommendation on specific surgical techniques and clinical practice.

### Strengths and limitations

Our systematic review, being the most inclusive to date, includes the largest number of RCTs on both entropion and ectropion. Comprising mainly of high-quality RCTs assessed with the Cochrane Risk-of-Bias tool (ROB2) ensures robust evidence. We avoided language restrictions to include all relevant trials in languages other than English. However, the study has limitations, notably the modest sample sizes in most included studies and inherent biases in the study design, warranting cautious interpretation of findings.

## Conclusion

Various modifications to the Lateral Tarsal Strip effectively treat lower eyelid malposition with high success rates, patient satisfaction, and low recurrence rates. Promising alternative treatments include Everting Sutures, Retraction Pelication, and Orbicularis Muscle Shortening, considering the variability in presentation. Individualized treatment based on patient characteristics is crucial. Further research is needed to refine the indications for each treatment modality.

## Authors’ contributions

Conceptualization: Lana Sbitan, Cristina Pires Camargo. Data curation: Lana Sbitan, Haneen Tanous, Mira Nawfal Jardak, Cristina Pires Camargo. Methodology: Cristina Pires Camargo. Project administration: Lana Sbitan. Resources: Lana Sbitan. Supervision: Cristina Pires Camargo. Validation: Cristina Pires Camargo. Visualization: Lana Sbitan. Writing-original draft: Lana Sbitan, Haneen Tanous, Mira Nawfal Jardak, Cristina Pires Camargo. Writing-review & editing: Lana Sbitan, Haneen Tanous, Cristina Pires Camargo.

## Abbreviations

MRD, Margin Reflex Distance; SS, Scleral Show; HLL, Horizontal Lid Laxity; LLE, Lower Lid Excursion; LTS, Lateral Tarsal Strip; ES, Everting Sutures; RP, Retractor Plication; OMS, Orbicularis Muscle Shortening.

## Conflicts of interest

The authors declare no conflicts of interest.

## Data Availability

The data and data extraction form is available from the corresponding author upon reasonable request.

## Appendix Supplementary materials

Image, application 1

## References

[1] Guthrie A.J., Kadakia P., Rosenberg J. (2019). Eyelid malposition repair: a review of the literature and current techniques. Semin Plast Surg.

[2] Pereira M.G.B., Rodrigues M.A., Rodrigues S.A.C. (2010). Eyelid entropion. Semin Ophthalmol.

[3] López-García J.S., García-Lozano I., Giménez-Vallejo C., Jiménez B., Sánchez Á., de Juan I.E. (2017). Modified lateral tarsal strip for involutional entropion and ectropion surgery. Graefes Arch Clin Exp Ophthalmol Albrecht Von Graefes Arch Klin Exp Ophthalmol..

[4] The PRISMA 2020 statement: an updated guideline for reporting systematic reviews | The BMJ [Internet]. [cited 2024 Jan 28]. Available from: https://www.bmj.com/content/372/bmj.n71.

[5] RoB2: A revised Cochrane risk-of-bias tool for randomized trials | Cochrane Bias [Internet]. [cited 2024 Feb 3]. Available from: https://methods.cochrane.org/bias/resources/rob-2-revised-cochrane-risk-bias-tool-randomized-trials.

[6] Altieri M., Iester M., Harman F., Bertagno R., Capris P., Venzano D. (2003). Comparison of three techniques for repair of involutional lower lid entropion: a three-year follow-up study. Ophthalmol J Int Ophtalmol Int J Ophthalmol Z Augenheilkd.

[7] Altieri M., Kingston A.E.H., Bertagno R., Altieri G. (2004). Modified retractor plication technique in lower lid entropion repair: a 4-year follow-up study. Can J Ophthalmol J Can Ophtalmol.

[8] Dulz S., Green S., Mehlan J., Schüttauf F., Keserü M. (2019). A comparison of the lateral tarsal strip with everting sutures and the Quickert procedure for involutional entropion. Acta Ophthalmol (Copenh).

[9] Goel R., Sanoria A., Kumar S., Arya D., Nagpal S., Rathie N. (2017). Comparison of polypropylene sling with combined transconjunctival retractor plication and lateral tarsal strip for correction of involutional lower eye lid ectropion. Open Ophthalmol J.

[10] Khan F.A., Hyder M.F., Khan Niazi S.P., Mirza U.T. (2021). Comparison of the recurrence rate of entropion via subciliary route and transconjunctival route in combined lateral tarsal strip with retractor advancement procedure for involutional entropion correction. J Coll Physicians Surg–Pak JCPSP..

[11] Meduri A., Inferrera L., Oliverio G.W., Tumminello G., Rechichi M., Mazzotta C. (2018). The use of a double suture and conjunctival cuts in the lateral tarsal strip: a new approach to involutional ectropion. J Craniofac Surg.

[12] Nakos E.A., Boboridis K.G., Kakavouti-Doudou A.A., Almaliotis D.D., Sioulis C.E., Karampatakis V.E. (2019). Randomized controlled trial comparing everting sutures with a lateral tarsal strip for involutional lower eyelid entropion. Ophthalmol Ther.

[13] Olver J.M., Rose G.E., Khaw P.T., Collin J.R. (1998). Correction of lower eyelid retraction in thyroid eye disease: a randomized controlled trial of retractor tenotomy with adjuvant antimetabolite versus scleral graft. Br J Ophthalmol.

[14] Scheepers M.A., Singh R., Ng J., Zuercher D., Gibson A., Bunce C. (2010). A randomized controlled trial comparing everting sutures with everting sutures and a lateral tarsal strip for involutional entropion. Ophthalmology.

[15] Xu Q. (2015). Clinical efficacy comparison of flabby skin excision combined orbicularis oculi muscle shortening surgery in patients with senile entropion. Guo Ji Yan Ke Za Zhi.

[16] Yuan W. (2020). Treatment of degenerative entropion with lower eyelid muscle reduction and lateral tarsal plate fixation. Int Eye Sci.

[17] Lateral eyelid block excision versus lateral tarsal strip procedure. | Dutch Trial Register [Internet]. [cited 2024 Jan 26]. Available from: https://onderzoekmetmensen.nl/en/trial/20295.

[18] ELhamaky T.R. mohamed M. Lower Eyelid Retractors Plication, Transcutaneous Blepharoplasty and Wedge Resection for Treatment of Lower Eyelid Involutional Entropion and Dermatochalasis [Internet]. clinicaltrials.gov; 2021 Jan [cited 2024 Jan 1]. Report No.: NCT04720586. Available from: https://clinicaltrials.gov/study/NCT04720586.

[19] ISRCTN - ISRCTN29030032: Lateral tarsal strip and everting sutures vs lateral tarsal strip and Jones procedure for involutional entropion: 2-year prospective randomized controlled trial [Internet]. [cited 2024 Jan 26]. Available from: https://www.isrctn.com/ISRCTN29030032.

[20] Chan D., Sokoya M., Ducic Y. (2017). Repair of the malpositioned lower lid. Facial Plast Surg FPS.

[21] Congenital eversion of upper eyelids: Case report and manage: Indian Journal of Ophthalmology [Internet]. [cited 2024 Feb 4]. Available from: https://journals.lww.com/ijo/fulltext/2006/54030/congenital_eversion_of_upper_eyelids__case_report.14.aspx.

[22] Hahn S., Desai S.C. (2016). Lower Lid Malposition: Causes and Correction. Facial Plast Surg Clin N Am.

[23] Hakim F., Phelps P.O. (2020). Entropion and ectropion. Dis–Mon DM..

[24] Boboridis K.G., Bunce C. (2011). Interventions for involutional lower lid entropion. Cochrane Database Syst Rev.

