# Supplementary material for: Towards optimal management of lower eyelid malpositions: A systematic review of treatment effectiveness and safety
Source: Clinics (Sao Paulo). 2024 Dec 3;80:100547. doi: 10.1016/j.clinsp.2024.100547 (PMC11652735; doi:10.1016/j.clinsp.2024.100547)
Supplement: Supplementary file 1 [file mmc1.docx]

**CLINICS-D-24-01163 – Supplementary Material**

**Supplementary Table 1** Quality Assessment of included clinical trials utilizing (RoB V2) quality assessment tool

| **Study ID** | **Olver et al. 1998**[1**]** | **Altieri et al. 2003**[2**]** | **Altieri et al. 2004**[3**]** | **Scheepers et al. 2010**[4**]** | **Xu, 2015** [5**]** | **López-García et al. 2017**[6**]** | **Goel et al. 2017**[7**]** | **Meduri et al. 2018**[8**]** | **Nakos et al. 2019**[9**]** | **Dulz et al. 2019**[10**]** | **Yuan, 2020**[11**]** | **Khan et al. 2021**[12**]** |
| --- | --- | --- | --- | --- | --- | --- | --- | --- | --- | --- | --- | --- |
| Randomization | Low Risk | Low Risk | Low Risk | Low Risk | Low Risk | Low Risk | Low Risk | Unclear Risk | Low Risk | Low Risk | Low Risk | Unclear Risk |
| Deviations from intended interventions | Low Risk | Unclear Risk | Unclear Risk | Low Risk | Low Risk | Low Risk | High Risk | High Risk | Low Risk | Low Risk | Unclear Risk | Low Risk |
| Missing outcome data | Low Risk | Low Risk | Low Risk | Low Risk | Low Risk | Low Risk | Low Risk | Unclear Risk | High Risk | Low Risk | Low Risk | Low Risk |
| Measurement of the outcome | Unclear Risk | Unclear Risk | Unclear Risk | Unclear Risk | Low Risk | Unclear Risk | Low Risk | Low Risk | Unclear Risk | Low Risk | Unclear Risk | Low Risk |
| Selection of the reported results | Low Risk | Low Risk | Low Risk | Low Risk | Low Risk | High Risk | Low Risk | High Risk | High Risk | Low Risk | Low Risk | Low Risk |
| Overall Bias | **Low Risk** | **Unclear Risk** | **Unclear Risk** | **Low Risk** | **Low Risk** | **High Risk** | **High Risk** | **High Risk** | **High Risk** | **Low Risk** | **Unclear Risk** | **Low Risk** |

1. Olver JM, Rose GE, Khaw PT, Collin JR. Correction of lower eyelid retraction in thyroid eye disease: a randomised controlled trial of retractor tenotomy with adjuvant antimetabolite versus scleral graft. Br J Ophthalmol. 1998;82(2):174-80.

2. Altieri M, Iester M, Harman F, Bertagno R, Capris P, Venzano D, et al. Comparison of three techniques for repair of involutional lower lid entropion: a three-year follow-up study. Ophthalmol J Int Ophtalmol Int J Ophthalmol Z Augenheilkd. 2003;217(4):265-72.

3. Altieri M, Kingston AEH, Bertagno R, Altieri G. Modified retractor plication technique in lower lid entropion repair: a 4-year follow-up study. Can J Ophthalmol J Can Ophtalmol. 2004;39(6):650-5.

4. Scheepers MA, Singh R, Ng J, Zuercher D, Gibson A, Bunce C, et al. A randomized controlled trial comparing everting sutures with everting sutures and a lateral tarsal strip for involutional entropion. Ophthalmology. 2010;117(2):352–5.

5. Xu QL. Clinical efficacy comparison of flabby skin excision combined orbicularis oculi muscle shortening surgery in patients with senile entropion. Int Eye Sci. 2015;15:1277-9.

6. López-García JS, García-Lozano I, Giménez-Vallejo C, Jiménez B, Sánchez Á, de Juan IE. Modified lateral tarsal strip for involutional entropion and ectropion surgery. Graefes Arch Clin Exp Ophthalmol Albrecht Von Graefes Arch Klin Exp Ophthalmol. 2017;255(3):619-25.

7. Goel R, Sanoria A, Kumar S, Arya D, Nagpal S, Rathie N. Comparison of Polypropylene Sling with Combined Transconjunctival Retractor Plication and Lateral Tarsal Strip for Correction of Involutional Lower Eye Lid Ectropion. Open Ophthalmol J. 2017;11:285-97.

8. Meduri A, Inferrera L, Oliverio GW, Tumminello G, Rechichi M, Mazzotta C, et al. The Use of a Double Suture and Conjunctival Cuts in the Lateral Tarsal Strip: A New Approach to Involutional Ectropion. J Craniofac Surg. 2018;29(8):2312-5.

9. Nakos EA, Boboridis KG, Kakavouti-Doudou AA, Almaliotis DD, Sioulis CE, Karampatakis VE. Randomized Controlled Trial Comparing Everting Sutures with a Lateral Tarsal Strip for Involutional Lower Eyelid Entropion. Ophthalmol Ther. 2019;8(3):397-406.

10. Dulz S, Green S, Mehlan J, Schüttauf F, Keserü M. A comparison of the lateral tarsal strip with everting sutures and the Quickert procedure for involutional entropion. Acta Ophthalmol (Copenh). 2019;97(6):e933-6.

11. Yuan W. Treatment of degenerative entropion with lower eyelid muscle reduction and lateral tarsal plate fixation. Int Eye Sci. 2020;2181-4.

12. Khan FA, Hyder MF, Khan Niazi SP, Mirza UT. Comparison of the Recurrence Rate of Entropion via Subciliary Route and Transconjunctival Route in Combined Lateral Tarsal Strip with Retractor Advancement Procedure for Involutional Entropion Correction. J Coll Physicians Surg Pak. 2021;30(4):429-33.

**Supplementary Table 2** Overview of ongoing clinical trials examining various treatments for lower eyelid malposition.

| **Title** | **Country** | **Study Design** | **Registration Date** | **Intervention** | **Control** | **Inclusion Criteria** | **Exclusion Criteria** |
| --- | --- | --- | --- | --- | --- | --- | --- |
| Lateral Tarsal Strip and Everting Sutures vs. Lateral Tarsal Strip and Procedure for Involutional Entropion: 2-year Prospective Randomized Controlled Trial[1] | United Kingdom | Randomized controlled trial | 9/28/2007 | Lateral tarsal strip and everting sutures | Lateral tarsal strip and Jones procedure | 1. Those over 60 with primary involutional lower eyelid entropion. | Not Available |
|  |  |  |  |  |  | 2. Suitable for local anesthesia. |  |
|  |  |  |  |  |  | 3. Capable of providing informed consent. |  |
| Lateral Eyelid Block Excision Versus Lateral Tarsal Strip Procedure[2] | Netherlands | Non-randomized controlled trial | 2/2/2009 | Lateral eyelid block excision | Lateral tarsal strip procedure | Individuals with eyelid conditions, such as ectropion, entropion, facial palsy, and eyelid laxity from ocular prosthesis use, planned to undergo a surgical procedure involving lateral horizontal eyelid tightening. | 1. Individuals under 18. |
|  |  |  |  |  |  |  | 2. Surgical procedures with both medial and lateral horizontal eyelid tightening. |
|  |  |  |  |  |  |  | 3. Eyelid malposition due to cicatricial diseases. |
|  |  |  |  |  |  |  | 4. Aesthetic blepharoplasty. |
| Surgical Treatment of Lower Eyelid Involutional Entropion and Dermatochalasis[3] | United Arab Emirates | Randomized clinical trial | 1/22/2021 | Lower eyelid blepharoplasty, retractor plication and wedge resection | Lower eyelid blepharoplasty and retractor plication | 1. 18 years and older (Adult, Older Adult) | 1. Other eyelid disease |
|  |  |  |  |  |  | 2. Lower eyelid involutional entropion | 2. Other causes of entropion |
|  |  |  |  |  |  | 3. Dermatochalasis | 3. Previous eyelid surgery |

1. ISRCTN - ISRCTN29030032: Lateral tarsal strip and everting sutures vs. lateral tarsal strip and Jones procedure for involutional entropion: 2-year prospective randomized controlled trial [Internet]. [cited 2024 Jan 26]. Available from: https://www.isrctn.com/ISRCTN29030032

2. Lateral eyelid block excision versus lateral tarsal strip procedure. | Dutch Trial Register [Internet]. [cited 2024 Jan 26]. Available from: https://onderzoekmetmensen.nl/en/trial/20295.

3. ELhamaky TR Mohamed M. Lower Eyelid Retractors Plication, Transcutaneous Blepharoplasty and Wedge Resection for Treatment of Lower Eyelid Involutional Entropion and Dermatochalasis [Internet]. clinicaltrials.gov; 2021 Jan [cited 2024 Jan 1]. Report No.: NCT04720586. Available from: https://clinicaltrials.gov/study/NCT04720586.
